# A Locally Advanced NSCLC Patient Harboring a Rare KIF13A-RET Fusion Benefited from Pralsetinib: A Case Report

**DOI:** 10.3390/curroncol31070281

**Published:** 2024-06-30

**Authors:** Zenghao Chang, Tengfei Zhu, Hao Jiang, Wei Ou, Siyu Wang

**Affiliations:** Department of Thoracic Surgery, Sun Yat-sen University Cancer Center, State Key Laboratory of Oncology in South China, Collaborative Innovation Center for Cancer Medicine, Guangzhou 510062, China; changch@sysucc.org.cn (Z.C.); zhutf@sysucc.org.cn (T.Z.); jianghao@sysucc.org.cn (H.J.); ouwei@sysucc.org.cn (W.O.)

**Keywords:** RET fusion, pralsetinib, adjuvant treatment, ctDNA, NSCLC, case report

## Abstract

The application of adjuvant treatment has significantly enhanced the survival of patients with resectable non-small cell lung cancer (NSCLC) carrying driver gene mutations. However, adjuvant-targeted therapy remains controversial for some NSCLC patients carrying rare gene mutations such as RET, as there is currently a lack of confirmed randomized controlled trials demonstrating efficacy. In this report, we describe the case of a 58-year-old man with stage IIIA NSCLC who underwent complete lobectomy with selective lymph node dissection. Postoperative next-generation sequencing revealed that the patient harbored a rare KIF13A-RET fusion. The patient elected to receive adjuvant treatment with pralsetinib monotherapy and underwent serial circulating tumor DNA (ctDNA) monitoring after surgery. During follow-up, despite experiencing dose reduction and irregular medication adherence, the patient still achieved a satisfactory disease-free survival (DFS) of 27 months. Furthermore, ctDNA predicted tumor recurrence 4 months earlier than imaging techniques. The addition of bevacizumab to the original regimen upon recurrence continued to be beneficial. Pralsetinib demonstrated promising efficacy as adjuvant therapy, while ctDNA analysis offered a valuable tool for early detection of tumor recurrence. By leveraging targeted therapies and innovative monitoring techniques, we aim to improve outcomes and quality of life for NSCLC patients in the future.

## 1. Introduction

Lung cancer remains one of the most frequently diagnosed cancers and continues to be the leading cause of cancer-related mortality worldwide, with non-small cell lung cancer (NSCLC) accounting for the majority of cases [1]. Despite significant advances in targeted therapies against driver genes such as EGFR, ALK and ROS1, in recent years, there remains a subset of patients with rare gene mutations who do not achieve adequate treatment responses. RET (rearranged during transfection) fusions have been identified in 1–2% of NSCLC patients, presenting as actionable targets [2]. Patients harboring RET fusion-positive tumors have shown modest clinical benefits from treatment with multikinase inhibitors, such as cabozantinib and vandetanib.

Pralsetinib, as a highly selective RET inhibitor, has been demonstrated to exhibit significant antitumor activity in advanced RET fusion-positive NSCLC patients [3,4]. However, clinical data regarding pralsetinib monotherapy for postoperative NSCLC with RET gene mutations remain limited. Furthermore, with the development of ctDNA technology, its potential value in monitoring disease recurrence and treatment response has also gained attention. 

Therefore, we present this case report describing a stage IIIA NSCLC patient who underwent adjuvant therapy with pralsetinib monotherapy following curative surgical resection, along with the utilization of ctDNA dynamic monitoring for disease progression. Our case report aims to explore the efficacy and safety of pralsetinib in NSCLC patients, as well as the potential role of ctDNA monitoring in guiding treatment, providing new insights and implications for clinical practice.

## 2. Case Presentation

**Figure 1:**
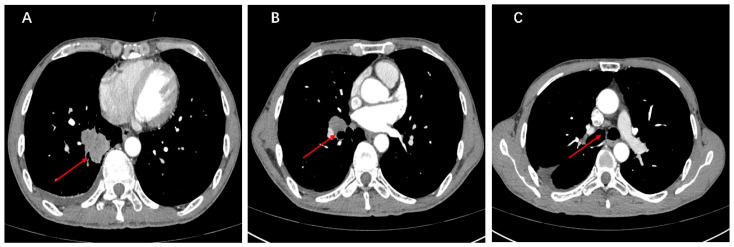
(**A**–**C**) Preoperative CT of the chest revealed a space-occupying lesion in the lower lobe of the right lung.

A 58-year-old Chinese male patient was admitted to the Sun Yat-sen University Cancer Center in March 2021, complaining of a persistent cough for two weeks, having smoked for 38 years. Chest computed tomography (CT) revealed a solid mass measuring 40 mm × 30 mm in the lower lobe of the right lung. Enlarged ipsilateral hilar and mediastinal lymph nodes were identified. There was a small amount of pleural effusion in the right chest cavity (Figure 1A–C). The pathology report from the CT-guided lung mass biopsy indicated NSCLC. The cytological examination of pleural effusion revealed no malignant tumor cells. There was no evidence of brain metastases radiographically based on a preoperative brain MRI, and a stage of cT2aN2M0 was determined. Following the completion of relevant preoperative evaluations, the patient underwent curative right lower lobectomy and mediastinal lymph node dissection on 9 March 2021. Pathological examination revealed metastasis to one hilar lymph node, one subcarinal lymph node, and one para-bronchial lymph node. The tumor tissue exhibited poorly differentiated infiltrating adenocarcinoma morphology with spread through airway space (STAS). The gross pathology examination indicated the tumor size was 45 mm × 38 mm × 35 mm, leading to an upgrade in staging to pT2b. According to the 8th edition of the AJCC (American Joint Committee on Cancer) staging system, the disease was diagnosed as stage IIIA adenocarcinoma of the lung (pT2bN2M0).

**Figure 2:**
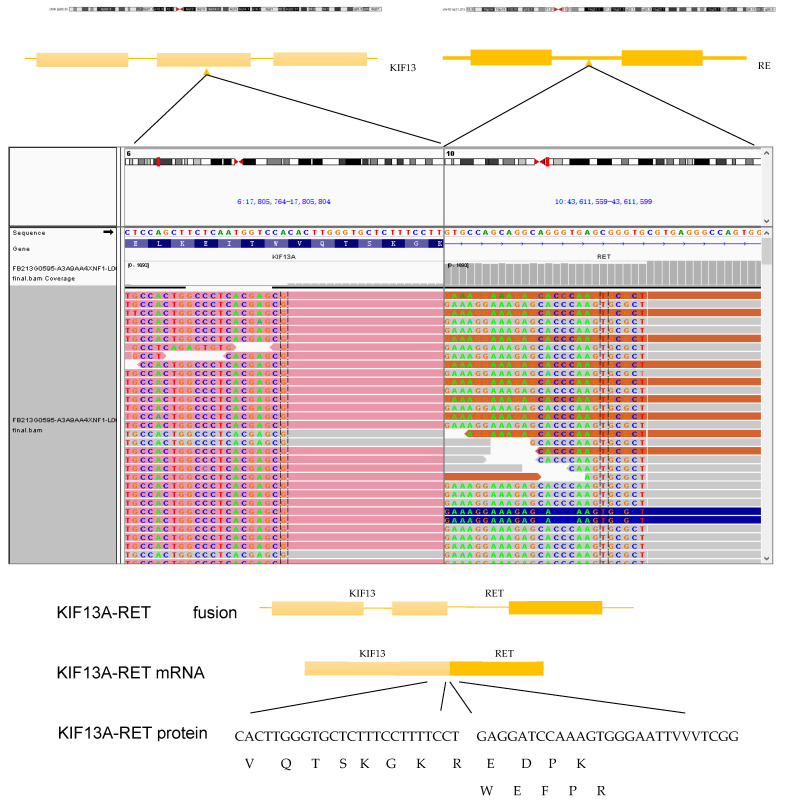
Tumor tissue NGS showed a rare KIF13A-RET fusion involving exon 19 of KIF13A and exon 12 of RET.

**Figure 3:**
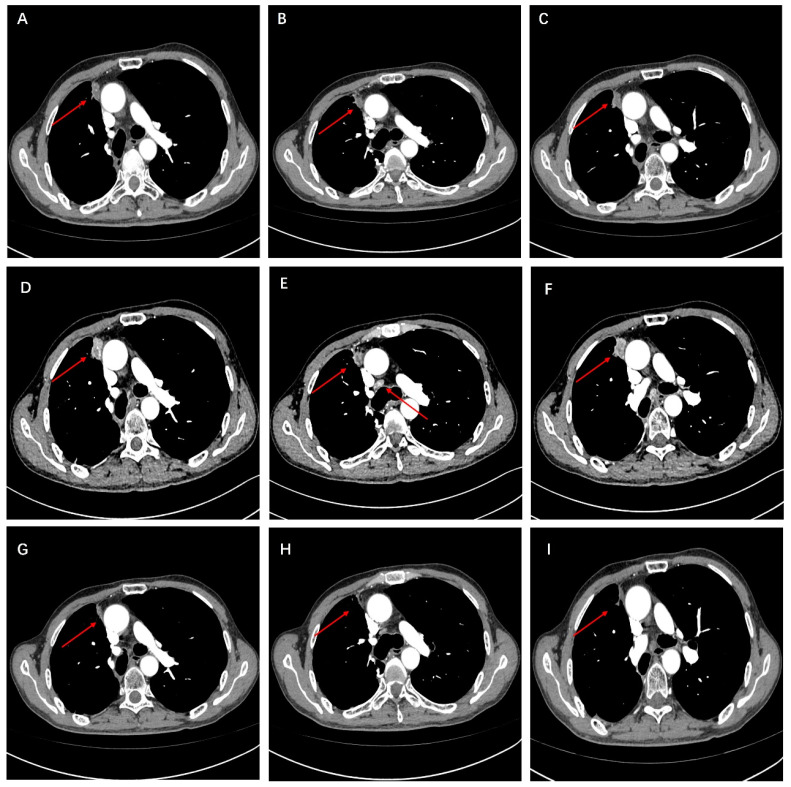
(**A**–**C**) At 27 months post-surgery, a follow-up chest-enhanced CT revealed nodules indicative of pleural metastasis on the right side. (**D**–**F**) After three months of continued treatment with the original regimen following recurrence, a follow-up CT scan revealed pleural metastatic lesions and enlarged mediastinal lymph nodes. (**G**–**I**) After three months of adding bevacizumab to the original regimen, a follow-up CT scan revealed a reduction in pleural metastatic lesions and shrinkage of mediastinal lymph nodes.

**Figure 4:**
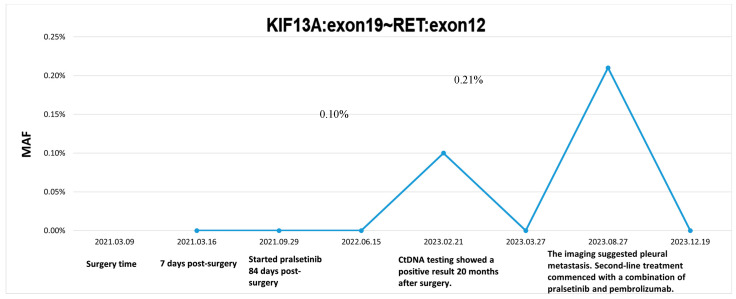
ctDNA dynamics throughout the clinical course.

Mutation profiling of ctDNA from peripheral plasma collected one week after surgery and DNA obtained from formalin-fixed paraffin-embedded (FFPE) tumor tissue sections was performed using next-generation sequencing (NGS). The NGS tests, which utilized the LungTrak panel, targeted 139 lung cancer-related genes and were conducted at a centralized, CLIA-certified, and CAP-accredited clinical testing center (Nanjing Geneseeq Technology Inc., Nanjing, China). The sequencing platform used for the study was Illumina. A rare KIF13A-RET fusion involving exon 19 of KIF13A and exon 12 of RET was detected in tumor tissue (Figure 2). Mutations in the TP53, APC and MYC genes were also detected in the tissue sample. CtDNA was not identified in the peripheral plasma one week after surgery. Considering the patient’s long history of smoking and tumor staging, postoperative therapy was recommended. However, the patient declined adjuvant chemotherapy. After further discussion and collaborative decision-making, the patient was started on oral pralsetinib at a dose of 400 mg/d on postoperative day 84. One month after starting the medication, the patient experienced adverse reactions such as fatigue and loss of appetite, prompting a dose adjustment of pralsetinib to 300 mg/d. Upon the patient’s request, ctDNA was monitored at periodic intervals using LungTrak ctDNA testing. The patient’s ctDNA levels, measured using the assay above-mentioned, 7 and 206 days after surgery, were both 0.0% mutation allele frequency (MAF), with repeat ctDNA analysis 12 months after taking pralsetinib indicating persistently negative ctDNA at 0.0% MAF. Due to personal compliance issues, the patient became irregular in taking medication after one year of adherence. In Feburary 2023, after 20 months on pralsetinib therapy, ctDNA assessment identified detectable KIF13A-RET fusion 0.1% MAF. The finding in ctDNA level prompted a chest and upper abdominal enhanced CT scan, which did not reveal any signs of recurrence or metastasis. After 1 month of regular pralsetinib intake, ctDNA test no longer detected the RET fusion. However, repeat CT imaging 3 months later revealed a single nodule in the right pleura, suggesting possible metastasis (Figure 3A–C). On 27 August 2023, ctDNA analysis identified detectable KIF13A-RET fusion 0.2% MAF again. Subsequent restaging imaging revealed growth in the right pleura nodules 3 month prior, and new enlargements were observed in the N4R and N7 nodes, consistent with disease progression (Figure 3D–F). In November 2023, two months after treatment with bevacizumab in conjunction with the original regimen, a follow-up chest CT scan revealed a reduction in both the pleural metastatic lesion and mediastinal lymph nodes compared to previous imaging (Figure 3G–I). Longitudinal ctDNA levels monitoring indicated a clearance to 0.0% MAF in December 2023, consistent with radiological changes. The results of dynamic monitoring of ctDNA during the treatment process are presented in Figure 4.

## 3. Discussion

The adjuvant therapy for NSCLC harboring RET fusion presents a clinical challenge due to the lack of confirmed randomized controlled trials (RCTs). Despite the recognized efficacy of targeted therapies in advanced RET-positive NSCLC, the optimal treatment strategy in the adjuvant setting remains uncertain. In this case report, we presented a patient with stage IIIA NSCLC harboring the KIF13A-RET fusion mutation who underwent surgery followed by adjuvant therapy with pralsetinib. The patient exhibited a favorable response to the treatment, achieving a disease-free survival (DFS) of 27 months, and continued to benefit from the addition of bevacizumab to the original regimen upon recurrence. To our knowledge, this is the first case documenting the utilization of a RET inhibitor for adjuvant therapy following surgical intervention. 

RET encodes a transmembrane receptor tyrosine kinase endowed with proto-oncogene properties [5]. Aberrant activation of the RET gene, including point mutations and gene fusions, serves as a potent oncogenic driver. RET gene fusions or rearrangements represent a major aberration in NSCLC, accounting for 1–2% of cases, with the most common fusion variants involving KIF5B-RET and CCDC6-RET [6]. It has been confirmed that chemotherapy and multikinase inhibitors (MKIs) had unsatisfactory efficacy for advanced RET-positive NSCLC patients [7]. A study of 74 patients with RET-rearranged lung cancer receiving checkpoint inhibitor therapy showed that the majority of patients had low PD-L1 expression levels and poorer responses to immunotherapy [8]. Another multicenter retrospective analysis revealed that RET-positive NSCLC patients treated with single-agent immunotherapy had a median progression-free survival (PFS) of only 2.1 months, suggesting that RET gene NSCLC patients may not benefit from adjuvant immunotherapy [9]. Currently, the adjuvant standard treatment regimen for early and locally advanced RET-positive NSCLC is still platinum-based doublet chemotherapy. However, the 5-year survival rate varies from 67% for stage IB disease to 39% for stage IIIA disease, with high rates of disease recurrence observed across all disease stages [10]. A multi-cohort, open-label, phase 1/2 ARROW clinical trial evaluated the efficacy and safety of pralsetinib treatment in RET-altered solid tumors, including NSCLC. The updated data from the ARROW study demonstrate an overall response rate (ORR) of 72% in treatment-naive advanced RET fusion-positive NSCLC patients (*n* = 75). In patients previously treated with platinum-based chemotherapy (*n* = 136), the ORR was 59%. And PFS was 13.0 and 16.5 months, respectively [4]. The ADAURA trial highlighted the effectiveness of targeted therapy in an adjuvant setting, providing a rationale for exploring similar approaches in RET-positive NSCLC. In patients with stage IIIA disease, the 5-year overall survival was 85% (95% CI, 76 to 91) in the osimertinib group compared to 67% (95% CI, 57 to 75) in the placebo group (HR for death, 0.37; 95.03% CI, 0.20 to 0.64; *p* < 0.001) [11]. Considering the significant DFS and overall survival (OS) benefits of osimertinib in adjuvant treatment for EGFR-positive patients demonstrated in the ADAURA trial, we attempted single-agent pralsetinib therapy for this stage IIIA patient. Compared to the median DFS of 14.4 months observed among stage IIIA patients in the control arm of the ADAURA study [12], this patient achieved a DFS of 27 months, indicating a positive impact of adjuvant pralsetinib for RET-positive NSCLC patients. Further prospective studies are needed to confirm the role of pralsetinib in adjuvant therapy for RET-positive NSCLC patients. As a non-invasive diagnostic tool, monitoring of ctDNA has become an important approach for early diagnosis, prognostic stratification, surveillance and treatment response assessment in NSCLC [13]. However, the clinical value of dynamic ctDNA monitoring in the comprehensive management of postoperative NSCLC patients still requires further investigation. An increasing body of literature supports the use of ctDNA monitoring for early detection of disease recurrence in postoperative NSCLC. In a prospective study by Li et al., 123 patients with resectable stage I to IIIA NSCLC were enrolled. Preoperative and postoperative plasma and tumor tissue samples underwent next-generation sequencing with a panel of 425 cancer-related genes. Blood samples were collected before surgery, postoperatively within 1 month, and every 3 to 6 months for up to 3 years. The study found that a ctDNA-positive status in the first postsurgical samples was linked to a shorter recurrence-free survival (RFS) (HR, 3.04; 95% CI, 1.22–7.58; *p* = 0.012). Furthermore, results from longitudinally collected plasma samples revealed that patients with longitudinal ctDNA positivity had significantly shorter RFS (HR, 3.46; 95% CI, 1.59–7.55; *p* < 0.001) and overall survival (OS) (HR, 9.99; 95% CI, 1.17–85.78; *p* = 0.010) compared to ctDNA-negative patients [14]. In the LUNGCA-2 study, which included 233 patients with stage I-III NSCLC, with a median follow-up of 35.9 months, the result showed that the postoperative recurrence risk for patients testing positive for dynamic MRD was 23.5 times higher compared to MRD-negative patients. Additionally, dynamic monitoring of ctDNA can provide earlier indications of tumor recurrence compared to imaging detection, with a median lead time of 273 days [15]. The use of ctDNA for dynamic monitoring of tumor recurrence showed promise in our case. CtDNA analysis provided real-time information about the molecular status of the tumor, allowing for early detection of recurrence. This is particularly valuable in cases where conventional imaging techniques may not detect minimal residual disease. Despite the promising applications of ctDNA analysis, it is important to acknowledge its limitations. The study by Li N et al. revealed that ctDNA was detectable preoperatively in only 24.8% of NSCLC patients with stage I to IIIA, highlighting the urgent need to improve the sensitivity of assays [14]. Additionally, not all genomic alterations are shed into the blood, which means ctDNA may not capture the full spectrum of tumor heterogeneity [16]. Another limitation of liquid biopsies is their inability to detect the phenotypic transformation of tumor tissue as a mechanism of treatment resistance and tumor progression. These factors underscore the necessity for further advancements in ctDNA detection technologies and the complementary use of tissue biopsies for comprehensive tumor profiling.

## 4. Conclusions

To our knowledge, this is the first report documenting the adjuvant use of pralsetinib in a patient with resected NSCLC harboring the rare KIF13A-RET fusion mutation. Our findings indicate that pralsetinib demonstrates potential efficacy in this setting, contributing to a satisfactory DFS of 27 months. Additionally, the use of ctDNA for dynamic monitoring enabled the early detection of potential recurrence, preceding imaging by 4 months. This case underscores the importance of incorporating innovative adjuvant targeted therapies and advanced monitoring techniques in managing rare genetic alterations in NSCLC. Personalized treatment strategies, guided by individual molecular profiles and continuous monitoring, are crucial for optimizing patient outcomes and maintaining quality of life. Future research should focus on validating these findings in larger cohorts and exploring combination therapies to further improve the efficacy of adjuvant treatment in NSCLC patients with rare genetic mutations.

## Data Availability

Data sharing is not applicable to this case report as no new data were created or analyzed in this study.
